# Epigenetics of Conjunctival Melanoma: Current Knowledge and Future Directions

**DOI:** 10.3390/cancers16213687

**Published:** 2024-10-31

**Authors:** Kaylea M. Flick, Hakan Demirci, F. Yesim Demirci

**Affiliations:** 1 Department of Human Genetics, School of Public Health, University of Pittsburgh, Pittsburgh, PA 15261, USA; 2 Department of Ophthalmology and Visual Sciences, University of Michigan, Ann Arbor, MI 48105, USA

**Keywords:** conjunctival melanoma, epigenetic, histone modification, DNA methylation, non-coding RNA, miRNA, lncRNA, circRNA, RNA modification, biomarkers

## Abstract

The purpose of this article is to provide a literature review of the epigenetic understanding of conjunctival melanoma (CM), with a primary focus on current gaps in knowledge and future directions in research. CM is a rare aggressive cancer that predominantly affects older adults. Local recurrences and distant metastases commonly occur in CM patients; however, their prediction and management remain challenging. Hence, there is currently an unmet need for useful biomarkers and more effective treatments to improve the clinical outcomes of these patients. Like other cancers, CM occurrence and prognosis are believed to be influenced by multiple genetic and epigenetic factors that contribute to tumor development/progression/recurrence/spread, immune evasion, and primary/acquired resistance to therapies. Epigenetic alterations may involve changes in chromatin conformation/accessibility, post-translational histone modifications or the use of histone variants, changes in DNA methylation, alterations in levels/functions of short (small) or long non-coding RNAs (ncRNAs), or RNA modifications. While recent years have witnessed a rapid increase in available epigenetic technologies and epigenetic modulation-based treatment options, which has enabled the development/implementation of various epi-drugs in the cancer field, the epigenetic understanding of CM remains limited due to a relatively small number of epigenetic studies published to date. These studies primarily investigated DNA methylation, ncRNA (e.g., miRNA or circRNA) expression, or RNA methylation. While these initial epigenetic investigations have revealed some potential biomarkers and/or therapeutic targets, they had various limitations, and their findings warrant replication in independent and larger studies/samples. In summary, an in-depth understanding of CM epigenetics remains largely incomplete but essential for advancing our molecular knowledge and improving clinical management/outcomes of this aggressive disease.

## Simple Summary

Conjunctival melanoma (CM) is an aggressive cancer with an unmet need for prognostic biomarkers and more effective treatments. In recent years, a rapid increase in available epigenetic technologies and epigenetic modulation-based treatment options has led to the development/implementation of various epi-drugs in the cancer field. Like other cancers, CM occurrence and prognosis are also believed to be influenced by multiple genetic and epigenetic factors. While the genetic understanding of CM has been significantly improved in recent decades, the epigenetic understanding of CM remains limited. A relatively small number of CM epigenetics studies published to date have revealed some potential biomarkers and/or therapeutic targets; however, their results warrant replication in independent and larger studies/samples. Furthermore, not all epigenetic aspects of CM have been investigated in published studies; hence, an in-depth understanding of CM epigenetics remains largely incomplete. Additional studies are therefore urgently needed to advance our epigenetic understanding of CM and improve clinical outcomes by taking advantage of new therapy options driven by epigenetic knowledge.

## 1. Background Information on Conjunctival Melanoma

Conjunctival melanoma (CM) is a rare cancer occurring on the surface of the eye. CM arises from melanocytes located in the basal layer of the conjunctival epithelium [1] and most commonly occurs in the bulbar conjunctiva [2], which is the part of the conjunctiva that is directly exposed to sunlight [3]. The patients affected by this cancer are predominantly of European descent and >50 years of age [4,5]. While some studies reported more common occurrence in men, others did not find significant sex-related differences [5,6,7,8,9,10]. CM is rare with incidence rate estimates ranging from 0.23 to 1.11 cases per million persons per year in Western countries [5,8]. While earlier studies (using data from before 2008) suggested a rising incidence of CM, more recent studies do not suggest the continuation of that trend, although geographical differences may exist [5,6,7,8,10,11]. Overall, the CM cases account for about 5% of all ocular melanoma cases and about 0.25% of all melanoma cases [12,13,14]. CM represents a biologically distinct type of melanoma that bears certain similarities to both sun-exposed cutaneous melanomas and other (non-sun-exposed) mucosal melanomas, as supported by the genetic changes that overlap with those seen in either (or both) of these two major melanoma types [15,16].

There are two known precursor conditions, conjunctival nevus and primary acquired melanosis (PAM) with atypia, which can lead to the development of CM. While PAM with atypia is responsible for up to about 75% of CM cases, the remaining arise from conjunctival nevi or de novo [2,14,17,18]. Conjunctival nevi are benign lesions that uncommonly increase the risk of developing CM [2,14,17,18]. De novo cases account for about 20% of all CM cases and are usually associated with less favorable outcomes [2,14,17,18].

CM is initially diagnosed by clinical examination and is subsequently confirmed by excisional biopsy and histopathological examination [19]. The current standard of care for CM involves local excision followed by adjuvant therapy such as focal cryotherapy, topical chemotherapy, or brachytherapy [1,19]. Advanced CMs may require orbital exenteration, which is an extensive and disfiguring surgical procedure [1,19]. CM is very prone to local spread/recurrence and distant metastasis through mainly the lymphatic but also vascular system [14]. Currently, 10 years after the initial CM diagnosis, the local recurrence rate is up to about 50% and the mortality rate is up to around 30% [20,21]. As suggested by these rates, a better understanding of CM development and progression (i.e., underlying molecular mechanisms) is warranted to inform future prevention and management strategies and help to improve clinical outcomes.

CM occurs sporadically, and like other sporadic cancers, its initiation and progression are believed to be influenced by multiple somatic genetic and epigenetic alterations. Similar to cutaneous and other mucosal melanomas, CM is characterized by overactive mitogen-activated protein kinase (MAPK) signaling (also known as RAS-RAF-MEK-ERK signaling) and/or phosphatidylinositol 3-kinase (P13K)-AKT-mTOR signaling pathway(s) [14,16,22,23]. These two biological pathways are responsible for transmitting the growth signals to the nucleus and altering the expression of multiple genes involved in cellular differentiation, proliferation, and survival [14,23]. The well-known CM-related genes involved in the MAPK and/or P13K-AKT-mTOR pathway(s) include the proto-oncogenes *BRAF*, *NRAS*, and *KIT*, and the tumor-suppressor genes *NF1* and *PTEN* (reviewed recently by Chang et al. [16]). In addition, the genes involved in telomere maintenance, including *TERT* and *ATRX*, are believed to play important roles in CM pathogenesis [16]. Maintenance of telomere length through TERT activation or ATRX loss helps cancer cells achieve immortality by preventing progressive telomere shortening in successive cell divisions. Furthermore, *ATRX* is known to contribute to epigenetic regulation, and several other newly implicated genes (pending confirmation in additional CM studies) also appear to play important roles in epigenetic mechanisms [16].

Genetic alterations observed in CMs include somatic mutations affecting the abovementioned genes/pathways as well as structural variations occurring throughout the genome. The UV mutational signature (often associated with high tumor mutational burden) is also frequently observed in CMs, especially if/when subjected to DNA damage caused by chronic sun exposure [16]. All these improvements in our understanding of CM genetics have opened the door to new systemic therapies for better CM management, such as targeted therapies for tumors with specific oncogenic mutations (e.g., BRAF/MEK inhibitors for tumors with *BRAF* mutations) and immunotherapies (e.g., for tumors with high mutational burden) [9,14,24,25,26]. Recent years have witnessed a rapid increase in available epigenetic technologies and epigenetic modulation-based treatment options, which has enabled the development/implementation of various epi-drugs with promising results in cancer therapies. In order to also take advantage of these novel therapeutic approaches for better CM management, an in-depth understanding of CM epigenetics is urgently needed but currently limited, as summarized in this review.

## 2. Background Information on Epigenetic Regulation Mechanisms

**Figure 1:**
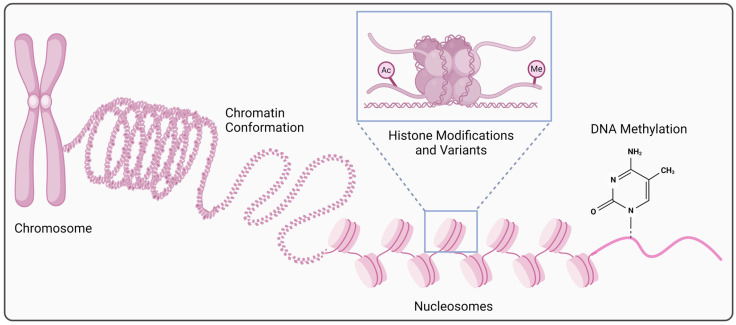
Epigenetic regulation at the transcriptional level. Alterations in chromatin structure and organization (conformation/accessibility), post-translational histone modifications (acetylation, methylation, etc.) or the use of histone variants, and changes in DNA methylation are involved in the cell type-, time-, or context-specific gene expression regulation at the transcriptional level (created in BioRender. https://BioRender.com/x40t223, accessed on 22 October 2024).

**Figure 2:**
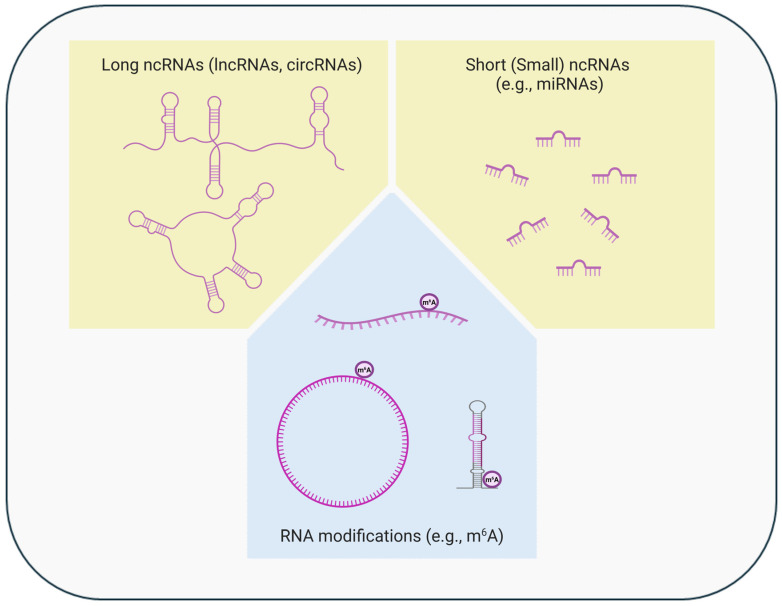
Epigenetic regulation at the post-transcriptional level. Alterations in levels/functions of long or short (small) non-coding RNAs (ncRNAs) and chemical RNA modifications (e.g., methylation) are involved in the cell type-, time-, or context-specific gene expression regulation at the post-transcriptional level (created in BioRender. https://BioRender.com/x92h200, accessed on 22 October 2024). The ncRNAs, especially the long ncRNAs and their modifications, can regulate gene expression also at the transcriptional level.

Epigenetics refers to the molecular mechanisms that regulate gene expression without altering the DNA sequence. Epigenetic regulation can occur either at the transcriptional or post-transcriptional level (Figure 1 and Figure 2), through alterations in chromatin conformation/accessibility, post-translational histone modifications or the use of histone variants, changes in DNA methylation, alterations in levels/functions of short (small) or long non-coding RNAs, or RNA modifications [27,28,29,30,31]. Various interactions, either synergistic or antagonistic, can also occur among these different epigenetic mechanisms [32,33,34,35,36]. Physiologically, these mechanisms are necessary for healthy development, maintenance of cell type/tissue-specific gene expression, and adaptive responses to internal and external changes/stimuli. In addition to gene expression regulation, epigenetic mechanisms are also important for DNA repair and replication [28,29,31,37,38,39]. Accordingly, accumulating evidence supports a major role of epigenetic alterations in tumor development/progression/recurrence/spread, tumor–immune system interactions, and response/resistance to cancer treatments [27,28,29,30,31,40,41,42,43,44,45]. Indeed, epigenetic reprogramming constitutes one of the hallmarks of sporadic cancer and is believed to be driven/influenced by accumulating genetic and epigenetic alterations and environmental exposures [46,47]. Due to their reversible and usually dynamic nature, epigenetic alterations represent attractive targets for new therapeutic options [27,30,45,48].

### 2.1. Chromatin Conformation and Accessibility

Genome activity is mediated by changes in the structure and conformation (spatial organization) of the chromatin, which is composed of nucleic acids and associated proteins [28]. Chromatin accessibility (closed/repressive, permissive/bivalent, or open/active chromatin) is regulated by the enzymes that are involved in DNA methylation or histone modifications, the proteins that can recognize these chemical modifications, and ATP-dependent remodeling complexes that can mediate the swapping of histones/variant histones into/out of nucleosomes or the (re)arrangement (sliding/repositioning or eviction) of nucleosomes [30,37,49,50,51]. All these chromatin modifiers (writers, erasers, and readers) play important roles in epigenetic regulation at the transcriptional level (Figure 1) and are often found mutated/altered in cancers [52,53].

### 2.2. Histone Modifications and Variants

Nucleosomes constitute the basic and repeating units of chromatin structure and organization (Figure 1). Each nucleosome is comprised of eight histone proteins (two copies of four core histones) and ~150 base pairs of the DNA double-wrapped around them [30,50,51]. Post-translational histone modifications can influence chromatin accessibility by affecting chromatin’s structure and compaction, leading to changes in its transcriptional state. These modifications (acetylation, methylation, as well as other alterations at specific amino acidic residues) predominantly occur at the N-terminal tail of the histones and can lead to either activation or repression of transcription by enabling or preventing the access of regulatory proteins to DNA elements [27,28,30,50,51]. Major enzyme groups responsible for the dynamic regulation of these modifications include “histone acetyltransferases (HATs), histone deacetylases (HDACs), histone methyltransferases (HMTs), and histone demethylases (HDMs)” [27,28,30,50,51]. Alterations in expression/function of these enzymes are often observed in cancers [52,54,55]. Histone variants, which differ from their canonical counterparts in their protein sequences, can replace the core histones in nucleosomes in specific genomic regions and contribute to genome integrity/stability [56,57,58].

### 2.3. DNA Methylation

DNA methylation typically involves the modification of cytosine (C) of Cytosine-phosphate-Guanine (CpG) dinucleotides by the addition of a methyl group to the 5-carbon position in the pyrimidine ring [5-methylcytosine (5-mC)] (Figure 1) [29,32,59]. DNA methylation is a repressive epigenetic mark as it leads to gene expression inhibition by attracting specific methyl-CpG-binding proteins for chromatin reconfiguration and preventing the transcription factors and other regulatory molecules from accessing the DNA [30,32]. Thus, “DNA methyltransferases (DNMTs) and methyl-binding domain (MBD) proteins” play major roles in transcriptional repression. While the majority of CpG sites are generally methylated in the human genome, the CpG islands (CpG-rich and mostly hypomethylated DNA sequences) are found in about 60–70% of gene promoters, of which the methylation state determines the gene activity status [29,59,60]. DNA methylation is usually a stable epigenetic mark as it leads to long-term gene silencing as related to its critical role in embryonic development, cell-type/tissue-specific gene regulation, genomic imprinting, X chromosome inactivation, and transposable elements silencing [30,32]. DNA demethylation can occur either passively (due to the failure of DNMT1 to maintain methylation through DNA replication) or actively (by removal of the methyl group from 5-mC through a chain of reactions catalyzed by TET proteins) [31,50,61,62,63]. An intermediary molecule generated during this chain of reactions is 5-hydroxymethylcytosine (5-hmC), the loss of which is implicated in cancer biology along with the mutations/dysregulation of TET genes/proteins [31,50,61,62,63]. Alterations in DNA methylation states are frequently observed in cancers including the focal promoter hypermethylation leading to tumor suppressor gene inactivation and the global hypomethylation leading to chromosomal instability, proto-oncogene activation, and drug resistance [29,59,64].

### 2.4. Non-Coding RNAs

Non-coding RNAs (ncRNAs) have mainly regulatory functions and are divided into two major groups: “(i) *short (small) ncRNAs (sncRNAs)* [microRNAs (miRNAs), short interfering RNAs (siRNAs), and P-element-induced wimpy testis (PIWI)-interacting RNAs (piRNAs)] and (ii) *long ncRNAs (lncRNAs)* [linear lncRNAs and circular RNAs (circRNAs)]” (Figure 2) [50,59,65,66,67,68].

Among the sncRNAs, the most studied are the miRNAs (~18 to 25 nucleotides in length), which are typically known to downregulate gene expression post-transcriptionally via degradation of their target mRNAs or repression of their translation [65,67]. A miRNA can target different mRNAs by recognizing and binding to their 3′ untranslated region (UTR), and the same mRNA can be targeted by different miRNAs [67]. Aberrantly expressed miRNAs can function as either onco-suppressors or oncomiRs when involved in oncogenesis [27,67]. MiRNA-mediated repression of tumor suppressor transcripts or lack of miRNA-mediated degradation of oncogenic transcripts can play active roles in cancers [27,50,65,67].

LncRNAs (>200 nucleotides in length) represent a large group of transcripts that can interact with various types of molecules (DNA, other RNAs, and proteins) and can *cis/trans*-regulate gene expression at the transcriptional and/or post-transcriptional level [27,29,65,69]. LncRNAs can also contribute to miRNA biogenesis or function as miRNA sponges [28,29,65]. As part of their involvement in cancer, dysregulated lncRNAs can act as tumor-suppressive or oncogenic molecules depending on their functions and interactions [65,67,70,71]. Non-coding circRNAs are covalently closed lncRNAs (their 5′ and 3′ ends are covalently linked by back-splicing), and they are involved in multiple biological processes similar to their linear counterparts; however, they are usually more stable due to their circular structure [28,65,67].

### 2.5. RNA Modifications

Both coding and non-coding RNAs can undergo diverse post-transcriptional chemical modifications; one of the most abundant being the methylation of adenosine residue at the N-6 position [N6-methyladenosine (m^6^A)], which can occur in 5′ and 3′ UTRs and internal long exons [30,51,72,73,74,75]. This modification can influence RNA processing/structure/function including its splicing, polyadenylation, stability, interactions, nuclear export, and translation [28,30,72,73]. The m^6^A modification is generated by N6-methyltransferases (the METTL3-METTL14 complex), removed by demethylases (ALKBH5 or FTO enzymes), and recognized by m^6^A-binding proteins [30,51,73]. Being studied increasingly, misregulated RNA modifiers (writers, erasers, and readers) and aberrant RNA modification profiles are emerging as important players in cancer biology (initiation, progression, spread, immune evasion, and resistance to therapy) [28,30,51,72,74,76,77,78].

## 3. Brief Overview of Melanoma Epigenetics

Epigenetic alterations are common in melanomas and can be influenced by both genetic and non-genetic factors affecting the tumor cells or their microenvironment. These alterations can contribute to melanoma initiation, progression, recurrence, and metastatic potential as well as the tumor’s interactions with the immune system and response to therapy [27,29,59,79]. Most of the current knowledge on melanoma-associated epigenetic alterations comes from the studies of cutaneous melanoma, as it represents the most common melanoma type. While various epigenetic mechanisms have increasingly been studied in melanomas, DNA methylation and miRNA expression are among the most frequently investigated.

*Chromatin structure/conformation* can be influenced by changes in levels/functions of the components of chromatin remodeling complexes, and altered chromatin remodeling can play an important role in melanomagenesis [29,59], as exemplified by the frequently dysregulated activity of the Switch/sucrose-non-fermentable (SWI/SNF) complexes. The components of these complexes are involved in DNA transcription, replication, repair, and genomic stability [80,81]. Of those components, the ones commonly altered in melanomas include the members of the ARID and SMARC protein families [80,81].

*Aberrant patterns of histone modifications* (e.g., hypoacetylation and hypermethylation) play active roles in melanomagenesis, mainly by silencing the tumor suppressor genes and leading to the dysregulation of signaling pathways, cell cycle progression, and apoptosis [29,59]. Consistent with these findings, altered levels/functions of histone modifier enzymes (e.g., various HDACs, HMTs, and HDMs) are often observed in melanomas [27,29,41,79,82]. Altered histone modifications can also influence the expression of genes that contribute to tumor immunogenicity and/or anti-tumor immunity within the tumor microenvironment [79]. *Aberrant expression of histone variants*, such as loss of macroH2A or overexpression of H2A.Z.2, have also been reported in correlation with melanoma progression as a result of cell cycle dysregulation and increased metastatic potential [29,59,83].

*DNA methylation aberrations* have frequently been studied in melanomas for their pathogenetic or prognostic influences. *Focal hypermethylation* of specific tumor suppressor gene promoters (e.g., *PTEN*, *CDKN2A*, and *APC* promoters) is commonly seen in melanomas, resulting in dysregulated intracellular signaling, cell cycle progression, apoptosis, and DNA repair [27,29,41,59]. In addition to known tumor suppressor genes, multiple other genes involved in cell differentiation/survival/growth or tumor immunogenicity and anti-tumor immunity have also been found aberrantly methylated in melanomas [29,59,79]. Some melanomas can exhibit a “CpG island methylator phenotype”, which is characterized by widespread CpG island hypermethylation associated with worse clinical outcomes [29,59,84,85]. Melanomas can also often show partial/complete *loss of 5-hmC*, which usually results from the deficiency of enzymes/cofactors (e.g., TETs) involved in active DNA demethylation [31,86,87]. Furthermore, melanomas can commonly exhibit *global DNA hypomethylation*, which can lead to genomic instability and oncogene activation [27,31].

*Aberrant expression of miRNAs and lncRNAs (linear lncRNAs or circRNAs)* has been increasingly studied in recent decades as related to melanoma pathogenesis and prognosis [27,41,59,88,89]. *Multiple miRNAs* have been found dysregulated in melanomas, leading to oncogenic, anti-apoptotic, and/or pro-metastatic properties, or influencing immune evasion and/or resistance to therapy [27,41,88,89]. Likewise, *several lncRNAs* have been found dysregulated in melanomas (e.g., *HOTAIR* and *MALAT1*), leading to oncogenic, anti-apoptotic, and/or pro-metastatic properties, or influencing immune evasion and/or drug resistance [27,41,88,89,90]. Melanoma-associated ncRNAs have therefore increasingly been investigated as potential biomarkers or new therapeutic targets [27,41].

*Aberrant m^6^A RNA methylation* has also been increasingly recognized for its involvement in melanoma, by influencing disease development/progression/metastasis, tumor immunogenicity or functions of tumor-infiltrating immune cells, and/or response to therapy [41,79,91]. Consistent with these observations, m^6^A-related modulators (writers, erasers, and readers) are frequently found altered in melanomas [91,92].

## 4. Current Knowledge of Conjunctival Melanoma Epigenetics

Only a handful of studies have been published to date concerning the epigenetic analyses of primary CMs, and they have mainly focused on *DNA methylation* [93,94], *ncRNA expression* (miRNAs or circRNAs) [95,96,97,98], or *RNA methylation* [99,100,101]. While chromatin conformation and histone modification analyses of CMs are currently lacking, alterations of the genes involved in the regulation of these processes have increasingly been implicated in CM, including altered chromatin remodelers (*ATRX* and *ARID2*) and altered histone modifiers (some HDAC, SETD, and KMT genes) [16].

As stated earlier and reviewed recently [16], CMs show genetic similarities to cutaneous and other mucosal melanomas, and the initial results from published CM epigenetics studies (reviewed below) similarly suggest an overlap between epigenetic alterations observed in CM and those reported in cutaneous and other mucosal melanomas.

### 4.1. DNA Methylation Studies of CM

A recent study by Jurmeister et al. [93] investigated the *DNA methylation* landscape of 107 melanomas originating from different primary sites (25 cutaneous and 82 non-cutaneous melanomas including 9 CMs) along with their genome-wide copy number analyses. Their results have revealed that conjunctival and other mucosal melanomas share a common *global DNA methylation* profile with cutaneous melanomas; however, the primary tumor sites differ in *promotor methylation* status and copy number changes of cancer-related genes. While the promoter methylation of tumor suppressor *APC* was the most common in CMs (6/9; 67%) compared to cutaneous (7/25; 28%) and other mucosal (8/63; 13%) melanomas, the promoter methylation of tumor suppressor *CDKN2A* was commonly present in both CMs (4/9; 44%) and cutaneous melanomas (9/25; 36%) but less commonly in other mucosal melanomas (6/63; 10%). The promoter methylation rate was comparable across these melanoma types for tumor suppressors *WIF1* (33–57%) and *RASSF1* (44–51%); however, it was relatively lower in CMs (11%) compared to cutaneous and other mucosal melanomas (24–33%) for tumor suppressor *PTEN*. The *CDKN2A* and *PTEN* tumor-suppressor genes were found to be inactivated in melanomas by either promoter methylation or focal deletion, the two events occurring in a mutually exclusive manner [93].

Given the molecular similarities between cutaneous and conjunctival melanomas, Stahl et al. [94] investigated whether CMs show a *global 5-hydroxymethylcytosine (5-hmC) loss* compared to the nevi, similar to that previously reported in cutaneous melanomas [86,87]. For this purpose, the authors performed immunohistochemistry and RNA in situ hybridization (RNA ISH) in 37 CMs and 40 conjunctival nevi to assess the expression of 5-mC, 5-hmC, and TET2 (a methylcytosine dioxygenase that catalyzes the conversion of 5-mC to 5-hmC). The loss of or reduction in 5-hmC has previously been linked to TET inactivation or downregulation [87]. Stahl et al. [94] were able to detect 5-hmC and TET2 in 54% and 35.1% of CMs and in 100% and 95% of conjunctival nevi, respectively, suggesting a significant loss of 5-hmC and TET2 occurring in CMs similar to cutaneous melanomas. While the levels/functions of TET proteins can be influenced by various factors, *TET2* mutations were identified in a subset of CMs analyzed by comprehensive genetic studies [15,102]. In addition, mutations in IDH genes, which can influence the functions of TET proteins [87,103], were also found in a subset of comprehensively analyzed CMs [15,104,105].

### 4.2. MicroRNA Expression Studies of CM

*MicroRNAs* are among the most studied epigenetic regulators in CM, investigated in three studies published to date [95,96,97]. In an initial 2016 study, Larsen et al. [97] performed a “microarray-based miRNA expression profiling” (for 2578 mature miRNAs) to compare archived [formalin-fixed, paraffin-embedded (FFPE)] CM samples (n = 37) with normal conjunctiva samples (n = 7). In addition, the authors tested the association of miRNA expression patterns with TNM stage, local recurrence, and distant metastases in CM. Their study identified 25 dysregulated (24 upregulated and 1 downregulated) miRNAs in CM compared to normal conjunctiva, including many miRNAs with known roles in cutaneous melanoma (most noteworthy being miR-20b-5p, miR-146a-5p, miR-146b-5p, miR-506-3p, and miR-509-3p). Furthermore, the authors were able to link seven upregulated miRNAs to the T stage and increased tumor thickness, and two upregulated miRNAs (miR-3687 and miR-3916) to local recurrence risk, but found no link to metastasis risk or mortality in CM. Moreover, when they analyzed/compared fresh frozen samples of CM (n = 6) with those of head and neck mucosal melanomas (n = 4), they found a resemblance in miRNA expression patterns. The authors thus concluded that CMs show a similar pattern of differentially expressed miRNAs compared to other melanomas, and some of the dysregulated miRNAs could potentially function as prognostic biomarkers or future therapeutic targets. In three patients, the authors were also able to compare fresh frozen and archived samples obtained from the same CMs and observe a good correlation in miRNA results, suggesting that archived (FFPE) samples could provide reliable results for miRNA expression analysis.

In a later study published by the same research group, Mikkelsen et al. [96] performed a “microarray-based miRNA expression profiling” of primary CMs with or without subsequent metastases to identify prognostic miRNAs associated with metastatic spread. The study analyzed archived (FFPE) samples from 25 patients with nonmetastatic CM and 13 patients with metastatic CM. When the authors compared primary CMs (with or without subsequent metastases) with normal conjunctiva, they identified several hundred differentially expressed miRNAs, including two (*miR-509-3p* and *miR-181b-5p*) similarly found upregulated in their previous study [97]. Pathway analysis of these two miRNAs linked them to Hippo and p53 signaling pathways as relevant to tumorigenesis. When the authors compared primary CMs with or without subsequent metastases, they discovered 15 differentially expressed miRNAs, including 9 upregulated and 6 downregulated in primary CMs with subsequent metastases. Of these 15 miRNAs associated with metastasis risk in CM, 4 (mir-575, mir-622, miR-1270, and miR-1290) were previously linked to other cancers. Furthermore, when the authors compared primary CMs with their pair-matched metastases in 13 patients, they detected 6 differentially expressed miRNAs, including 2 upregulated (miR-1246 and miR-302d-5p) and 4 downregulated (mir-6084, miR-184, mir-658, and mir-4427) in distant metastases, 3 of which (miR-1246, miR-184, and mir-658) were previously linked to other cancers. Using quantitative PCR (qPCR), the authors were able to confirm the downregulation of *miR-184* in metastatic vs. primary tumors from the same patients and nominate this extracellular matrix (ECM)-interaction pathway-related miRNA as a potential candidate for therapy. Pathway analysis of miRNAs dysregulated either in metastasis-prone primary CMs or in metastatic lesions linked these miRNAs to “cancers, cell growth and death, signal transduction, metabolism, and immune system”. While acknowledging their small sample size due to the rarity of CM, the authors concluded that the primary CMs with or without subsequent metastases show distinct global miRNA expression profiles, but larger studies are necessary to characterize the specific set of miRNAs predictive of metastatic potential in CM. Because of the poor correlation between the microarray and qPCR results, however, the authors could not recommend one method or another for future miRNA expression studies of CM.

In a more recent study from another research group, van Ipenburg et al. [95] used “TaqMan Low-Density Array Card A for miRNA expression profiling” to compare benign vs. malignant conjunctival melanocytic lesions, as well as the primary CMs with vs. without metastases, in order to identify the discriminating miRNAs between these study groups. Their study included a discovery cohort of 6 conjunctival nevi and 20 CMs and a replication cohort of 13 conjunctival nevi and 19 CMs. Both study cohorts were comprised of archived (FFPE) samples, and the study analysis primarily focused on miRNAs upregulated in CMs. While the authors found no miRNA expression differences between the primary CMs with vs. without metastases, they were able to identify and replicate a set of five miRNAs upregulated in primary CMs compared to the nevi, including “*miR-9-5p*, *miR-196b-5p*, *miR-450a-5p*, *miR-501-5p*, and *miR-615-3p*”. Three of these miRNAs (*miR-196b-5p*, *miR-615-3p*, and *miR-9-5p*) represented the best-performing miRNAs possibly involved in a shared pathway of homeobox gene clusters. The authors concluded that while further studies are needed to identify the miRNAs that could predict the metastatic potential of CM, their results suggest a potential benefit of miRNA profiling to discriminate benign vs. malignant lesions, especially in situations where the limited tissue availability can make such distinction difficult using routine methods.

### 4.3. CircRNA Expression Studies of CM

In a single study published to date on the role of *circRNAs* in CM, Shang et al. [98] performed RNA-seq to evaluate the expression of circRNAs in three CM samples compared to the paired adjacent normal tissues, and they identified a large number of potentially CM-relevant circRNA candidates. Upon further investigation with functional analyses, the authors characterized *CircMTUS1* (a circRNA derived from exons 2-3 of *MTUS1*) as an oncogenic circRNA upregulated in CM. *CircMTUS1* appeared to function as a miRNA sponge for *miR-622* and *miR-1208* to promote tumorigenesis by modulating several tumor-associated biological pathways (such as ErbB, MAPK, and Wnt signaling pathways). Both the host gene of this circRNA (*MTUS1*) and *miR-622* targeted by this circRNA are known to function as tumor suppressors in several cancers [106,107].

### 4.4. RNA Modification Studies of CM

Two recent studies [100,101] investigated the role of *RNA methylation (m^6^A)* in ocular melanomas by analyzing intraocular (uveal) and ocular surface (conjunctival) melanomas together. After evaluating 88 ocular melanoma and 28 normal melanocyte tissues using various analyses (including m^6^A assay in a subset; n = 14), Jia et al. [101] detected decreased levels of global m^6^A (associated with tumor progression) and reduced methylation of tumor-suppressor *HINT2* mRNA (hampering its translation efficiency) in ocular melanomas. Decreased global m^6^A levels appeared to be mediated by downregulation of m^6^A ‘writer’ METTL3 and upregulation of m^6^A ‘eraser’ ALKBH5, both associated with poor prognosis. The authors demonstrated that, in normal conditions, the methylation of *HINT2* mRNA facilitated its recognition and promotion for translation by YTHDF1, an m^6^A ‘reader’ protein with both m^6^A-containing RNA and ribosome binding activities. This observation provided a mechanistic insight into the role of m^6^A modification in tumorigenesis as a modulator of translation. In a later study that examined 47 ocular melanoma and 27 normal melanocyte tissues, He et al. [100] demonstrated the upregulation of *BACE2* mRNA, which appeared to be mediated by increased m^6^A RNA methylation. Upon further functional analysis, the authors found that the *BACE2* mRNA upregulation was associated with intracellular calcium release leading to ocular melanoma progression.

In a more recent study by Liao et al. [99], the authors studied 41 CM and 11 melanocytic nevi samples, and upon conducting multi-omic and functional analyses, they showed that the m^6^A ‘eraser’ *FTO* was upregulated in cancer-associated fibroblasts (CAFs) of the CM microenvironment that displayed pronounced proangiogenic potential (through the activation of VEGFA and EGR1). The authors demonstrated that the increased elimination of m^6^A modifications from *VEGFA* and *EGR1* by upregulated FTO prevented the YTHDF2-mediated mRNA decay, which in turn led to increased stability and upregulation of *VEGFA* and *EGR1*. Given the pivotal role of FTO in CAF-mediated angiogenesis and the critical role of angiogenesis in tumor progression and spread, the authors proposed FTO as a promising antiangiogenic therapeutic target to improve CM treatment. Interestingly, in addition to somatic FTO changes similarly shown to play a role in cutaneous melanomagenesis [108], recent genome-wide association studies have implicated germline *FTO* polymorphisms in genetic susceptibility to develop cutaneous melanoma [109,110,111].

## 5. Gaps in Knowledge of Conjunctival Melanoma Epigenetics and Future Directions

The emerging epi-drugs for cancer therapy (as a single agent or combined with other drugs/therapies) currently include those targeting chromatin remodelers (e.g., SMARC inhibitors), histone modifiers or readers (e.g., HDAC, HMT, HDM, or BET inhibitors), DNA methylation-related enzymes (e.g., DNMT inhibitors), ncRNAs (e.g., lncRNA or miRNA mimics or antagonists), or RNA-modifying enzymes (e.g., ALKBH5 or FTO inhibitors) [30,32,43,44,53,112,113,114,115]. The promising results from the latest clinical studies/trials on combination therapies (epi-drugs with other treatments) are especially encouraging given the commonly observed primary/acquired resistance to existing cancer therapies (immunotherapies, targeted therapies, or conventional therapies), as epigenetic reprogramming appears to play a crucial role in the development of such resistance [30,42,43,44,53,112,113]. Furthermore, some chemotherapy drugs (e.g., curaxins) that have been found to show their anti-cancer activity through chromatin damage rather than DNA damage (e.g., CBL0137 causing nucleosome destabilization and histone eviction) are currently tested in melanoma clinical trials as safer drug options [52]. Lastly, the recently emerging CRISPR-based targeted epigenetic editing tools provide exciting opportunities for future clinical interventions [116]. To be able to take advantage of these emerging drugs and therapeutic opportunities for better CM management, we first need to improve our understanding of epigenetic alterations contributing to CM development/progression and its microenvironment by conducting additional studies aiming to fill the gaps in our knowledge of the epigenetic makeup of CM.

Currently, epigenetic studies on chromatin conformation/structure and histone modifications are completely lacking in CM. A recent study [93] on DNA methylation described shared global but different promoter methylation patterns between CM and other mucosal and cutaneous melanomas; however, it included only nine CM samples and thus warrants replication in larger studies. Another study [94] reported a significant loss of 5-hmC and TET2 in 37 CMs vs. 40 conjunctival nevi, a finding that awaits confirmation in independent studies. A few studies that explored RNA methylation [99,100,101] or circRNAs [98] in primary CMs or their microenvironment have revealed some interesting findings, which again warrant replication in independent and larger studies. The studies on linear lncRNAs, on the other hand, are currently completely lacking in CM.

Three studies published on CM-related miRNA expression profiles (investigated by either microarray-based or TaqMan methods) [95,96,97] have revealed some dysregulated miRNAs, which were associated with CM development (compared to normal conjunctiva), CM recurrence or metastasis risk (prognostic potential), or discrimination of CMs from nevi (diagnostic potential); however, the findings were not very reproducible across studies or screening platforms. Methodological differences, the use of FFPE rather than fresh frozen tissues, and small sample sizes were proposed as potential factors that might have contributed to observed inconsistencies. Therefore, additional studies are needed using larger cohorts and employing the latest technologies, such as miRNA-seq, to further explore the miRNA dysregulation in CM for clinical biomarker and therapeutic target discovery. Moreover, investigating ‘circulating’ miRNAs in biofluids (e.g., in tear or serum/plasma samples) from CM patients could be an alternative and attractive strategy for biomarker discovery/development as compared to tissue-based analyses [95]. While the initial studies have looked at the miRNA expression differences between CM and nevi, studies on comparisons between CM and PAM (a precursor with a greater risk for malignant transformation) are currently lacking [95]. Since PAM with atypia is an important risk factor for CM, molecular characterization of predictive factors of PAM to CM transformation could have important implications for better follow-up and early treatment of CM patients [95]. There is also a need for more advanced-stage tumor-related research to identify differentially expressed miRNAs associated with advanced CM [97]. Identification of advanced CM-associated molecular factors could aid the prognostication efforts for better follow-up and timely treatment of CM patients [97].

## 6. Conclusions

CM is a rare but highly aggressive cancer. While local recurrences and distant metastases commonly occur in CM patients, their prediction and management remain difficult. Predictive biomarkers and more effective treatments are therefore urgently needed to help reduce the recurrence and metastasis rates and improve clinical outcomes in CM patients. CM is an age-related condition, and as the population ages and medical advances prolong the average lifespan, more CM cases may occur in the future and benefit from improved clinical management.

Like other cancers, CM initiation and progression are believed to be influenced by multiple genetic and epigenetic factors that contribute to tumorigenesis, recurrence/spread, immune evasion, and primary/acquired therapy resistance. While our genetic understanding of CM has been significantly improved in recent decades, our epigenetic understanding of CM remains largely incomplete due to a limited number of studies published to date. Recent years have witnessed a rapid increase in available epigenetic technologies and epigenetic modulation-based treatment options in the cancer field. In light of these advancements and emerging new therapies, an in-depth understanding of CM epigenetics has become essential to help improve the clinical outcomes in CM patients. Identifying epigenetic factors promoting CM development, progression, and spread can allow patients to receive more effective treatments through the use of epi-drugs, alone or in combination with other therapies, and lead to more favorable outcomes. Apart from the potential therapeutic benefits, advanced knowledge of CM epigenetics can also guide the diagnostic/prognostic biomarker development efforts for this aggressive disease and help implement more effective follow-up and timely treatment strategies.
